# UHPLC‐Q‐TOF‐MS/MS Dereplication to Identify Chemical Constituents of *Hedera helix* Leaves in Vietnam

**DOI:** 10.1155/2022/1167265

**Published:** 2022-08-08

**Authors:** Hong Ngoc Pham, Chieu An Tran, Thi Diep Trinh, Ngoc Lan Nguyen Thi, Huynh Nhu Tran Phan, Van Nhan Le, Ngoc Hung Le, Van Trung Phung

**Affiliations:** ^1^Center for Research and Technology Transfer, Vietnam Academy of Science and Technology (VAST), Hanoi 100000, Vietnam; ^2^Da Lat University, Dalat 66000, Lam Dong, Vietnam; ^3^Nguyen Tat Thanh University, Ho Chi Minh City 70000, Vietnam

## Abstract

*Hedera helix* has been reported to contain a wide range of metabolites and produce many pharmacological effects. This research demonstrates the determination and evaluation of the phytochemical profiling of *H. helix* grown in central Vietnam. Methanolic extract of ivy had been analyzed by ultra-high-performance liquid chromatography-quadrupole time-of-flight mass spectrometry (UHPLC‐Q‐TOF‐MS/MS). MS, and MS/MS experiments were manipulated using both negative and positive ionization modes to provide molecular mass information and production spectra for the structural elucidation of compounds. A total of 46 compounds including 24 triterpene saponins and other compounds were successfully identified of which four established saponin structures have been reported for the first time. This study has provided a base for building a quality control of the raw materials according to the profile of triterpene saponins and assessment of pharmaceutical ingredients of *H. helix* planted in Vietnam.

## 1. Introduction

*Hedera helix* L., the common ivy, is one of the 15 species of the genus *Hedera*, Araliaceae family. As an evergreen dioecious woody liana [1], ivy has an intense vitality, even in the cold winter. The common ivy is a popular ornamental plant in many countries. The plant grows naturally in Western, Central, and Southern Europe, North America, and Asia [2]. *Hedera helix* is applied to treat overactive thyroid (hyperthyroidism), rheumatic diseases, and respiratory tract inflammation [2]. In Vietnam, ivy is grown in areas with cool climates such as Da Lat, Moc Chau, and Sapa and is mainly used for decoration. Therefore, it has been little published research on its chemical composition and pharmacological effects. Therefore, the identification of chemical constituents is significant in the phytochemistry study of *H. helix* grown in Vietnam.

Phytochemical compounds were detected in ivy leaves including flavonoids, coumarins, phenolic acids, sterols, alkaloids, anthocyanins [3–5], and, in particular, triterpene saponins [1]. Some of the triterpene saponins and derivatives isolated from the ivy plant were as follows: helixoside A, helixoside B, 3-O-*β*-glucosyl hederagenin, 3-O-*β*-glucosyl-(1⟶2)- *β*-glucosyl oleanolic acid, 3-O-*β*-glucosyl-(1⟶2)-*β*-glucosyl hederagenin, 3-O-*β*-glucosyl 28-O-*β*-glucosyl-(1⟶6)-*β*-glucosyl hederagenin, hederagenin, oleanolic acid, bayogenin, *α*-hederin, hederagenin 3-O-*β*-glucoside, and hederasaponins B, C, D, E, F, G, H, and I [6].

A triterpene saponin's chemical structure is composed of an aglycone and sugar chain(s). In most cases, a series of saponins can be found in the same plant, with similar skeleton but slightly varied sugar chains. Due to the strong polarity and structural similarities, isolating a single saponin compound is frequently challenging. Furthermore, even with high-resolution nuclear magnetic resonance (NMR), structural elucidation of saponin is difficult, especially when the sugar chain contains more than three sugar residues [7]. As a result, a new method for quickly identifying and characterizing existing and novel structures is required.

UHPLC‐Q‐TOF‐MS/MS has been an increasingly powerful and important technique for elucidating chemical structures [8]. UHPLC-QTOF-MS/MS is capable of accurately measuring molecular mass by giving the elemental composition of obtained ions. The technique has been widely used in analyzing complex samples due to its high resolution and sensitivity. In some previously published research, UHPLC-QTOF-MS/MS was applied to characterize chemical constituents and metabolites in medicinal herbs, and obtained considerable results. Small metabolites profiling of the *Eurycoma longifolia* aqueous extracts were performed using LC-MS/MS [9]. An integrated approach using UHPLC-QTOF-MS/MS was developed for the systematic analysis of 46 physalins from the crude extracts of *Physalis alkekengi* calyx [10]. Sun et al. identified 31 saponins in *Shizhu ginseng* applying UPLC‐MS/MS [11]. UPLC‐Q‐TOF‐MS/MS‐guided dereplication of *Pulsatilla chinensis* was conducted, which resulted in the identification of 22 triterpenoid saponins (11 pairs of isomers) with four aglycone skeletons [12].

In addition, ivy leaves extracts were noted to exhibit antimicrobial, anthelmintic, antimutagenic, antitumor, antileishmanial, antithrombin, antioxidant, antispasmodic, and anti-inflammatory activities [13–21]. Triterpene saponins are the bioactive compounds responsible for the medicinal use of ivy [1]. Hederagenin has potential antitumor activity [22]. Hederasaponin B has antiviral activity against Enterovirus 71 subgenotypes C3 and C4a, via inhibiting the viral VP2 protein expression and blocking viral capsid protein synthesis [23]. *α*-hederin has a potent inhibitory effect on breast cancer cell development and promotes apoptosis in these cells [24]. Hederacoside C was reported to have anti-inflammatory effect against induced acute lung inflammation by *Staphylococcus aureus* both in vivo and in vitro [25].

To the best of our knowledge, the UHPLC‐Q‐TOF‐MS/MS study of phytochemicals in the *H. helix* leaves extract has not been announced. The present study aims to characterize the chemical constituents, especially triterpene saponins presented in the ivy leaves planted in Vietnam. The results of this work can assist in clarifying the metabolic profile of *H. helix*. Acceleration of finding the new compounds and assessment of the potential ingredients from this valuable species are concerned for pharmaceutical application.

## 2. Materials and Methods

### 2.1. Chemicals and Reagents

Deionized water for HPLC and HPLC grade acetonitrile, methanol, and analytical grade formic acid (≥98%) were obtained from Scharlau (Barcelona, Spain).

Two reference standards including *α*‐hederin and hederacoside C were obtained from Sigma-Aldrich Chemical Co. (Singapore). The purity of each compound was no less than 98%. The standards were stored at 4°C before being used for analysis.

### 2.2. Sample Preparation

*Hedera helix* was collected from Da Lat province, Vietnam, and identified by botanist Tran Huu Dang MSc, Southern Institute of Ecology, Vietnam Academy of Science and Technology. A voucher specimen (Code: NaPro.33.1019) was deposited in the Center for Research and Technology Transfer, Vietnam Academy of Science and Technology. The leaves were gently washed, allowed to air dry, and cut into fine pieces. 100.0 mg of leaves pieces was accurately weighed into a tube with a cover, and 2.0 mL of methanol-water (8 : 2, v/v) solvent was added. The sample was ultrasonicated for 10 min and then heated to 50°C for 5 min. After being centrifuged, the extract was pipetted to a 10.0 mL volumetric flask. The residue was continued on the extraction step. After five times of extraction, the solution was exactly scaled up to 10.0 mL using the solvent solution. The sample was filtrated through a 0.45-*μ*m filter membrane before injecting it for UHPLC‐Q‐TOF‐MS/MS analysis. Standard solutions of *α*‐hederin and hederacoside C were prepared in methanol at a concentration of 1000 (ppm).

### 2.3. UHPLC-Q-TOF Analysis

Sample analysis was performed on an ExionLC™ UHPLC system (AB SCIEX, USA) consisting of an ExionLC degasser, AC pumps, AC autosampler, controller, and AC column oven. Samples were analyzed on a Hypersil GOLD C18 column (150 × 2.1 mm, 3 *µ*) (Thermo Fisher Scientific, USA). The mobile phase, water containing 0.1% formic acid (A) and acetonitrile containing 0.1% formic acid (B), was run at a flow rate of 0.4 mL/min at 25°C. The gradient programming was as follows: 0–4 min, 2–20% B; 4–30 min, 20–68% B; 30–32 min, 68–98% B; 32–40 min, 98% B. Sample injection volume was 5.0 *μ*L.

An AB SCIEX X500R QTOF mass spectrometer (AB SCIEX, USA) with a Turbo V ion source was coupled with the UHPLC system. Mass data were acquired in both negative and positive electrospray ionization (ESI) modes. The MS conditions were set as follows: the ion source temperature, 500°C; curtain gas, 30 psi; nebulizer gas (GS 1), 45 psi; heater gas (GS 2), 45 psi. For the TOF MS scan, the mass range was set at *m*/*z* 70–2000. For the TOF MS/MS scan, the mass range was set at *m*/*z* 50–1500. For the negative mode, ion spray voltage was set at −4.5 kV, the declustering potential (DP) was −70 V, the collision energy (CE) was performed at −20 eV, and the collision energy spread (CES) was 10 eV. For the positive mode, the ion spray voltage was set at 5.5 kV, the DP was 80 V, the CE was 20 eV, and the CES was 10 eV.

**Figure 1:**
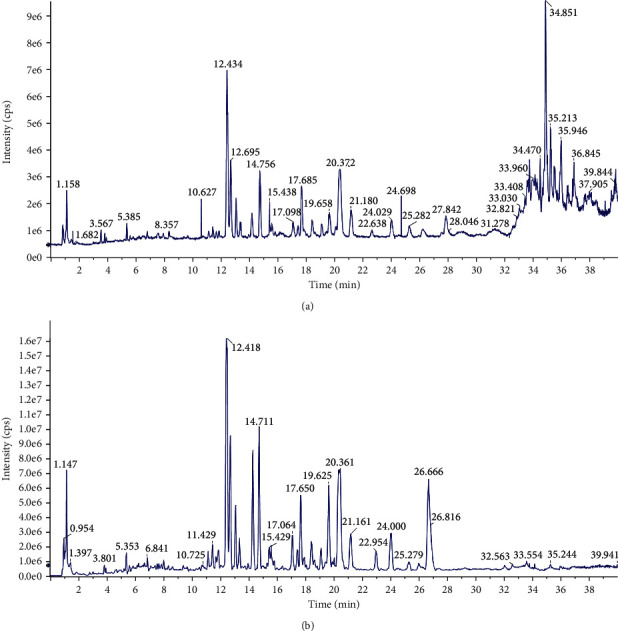
TIC of *Hedera helix* in (a) positive and (b) negative modes.

All the obtained data were processed by SCIEX OS software version 1.2.0.4122 (AB SCIEX, USA). The total ion chromatograms (TICs) of the *Hedera helix* extract in both positive and negative modes are shown in Figure 1.

## 3. Results and Discussion

### 3.1. Triterpene Saponins

#### 3.1.1. Aglycones

*Hedera helix* aglycones include hederagenin and oleanolic acid. A relatively abundant series of dehydrated ions and/or a small aglycone ion can readily distinguish the parent skeleton for the aglycone. In the positive mode, the diagnostic fragment ions of these two aglycones can be easily detected.

For the hederagenin‐type aglycone, the precursor ion [M+H]^+^ at *m*/*z* 473.3631 could produce two specific fragment ions at *m*/*z* 455.3525 and 437.3419 by the elimination of H_2_O. In many cases, the product ions at *m*/*z* 427.3576, 409.3470, and 391.3365 were observed by the losses of HCOOH from the three precursor ions mentioned earlier.

For the oleanolic‐type aglycone, the precursor ion [M+H]^+^ at *m*/*z* 457.3682 and the characteristic fragment ions at *m*/*z* 439.3576 by the neutral loss of H_2_O were observed. In addition, the product ions at *m*/*z* 411.3629 and 393.3522 were presented by the losses of HCOOH from the two predominant ions.

**Figure 2:**
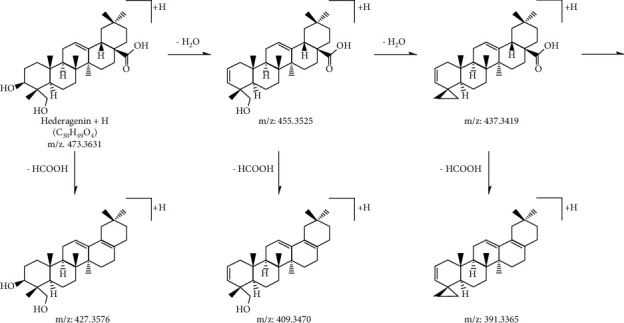
MS/MS fragmentation pathway of hederagenin aglycone in positive mode.

**Figure 3:**
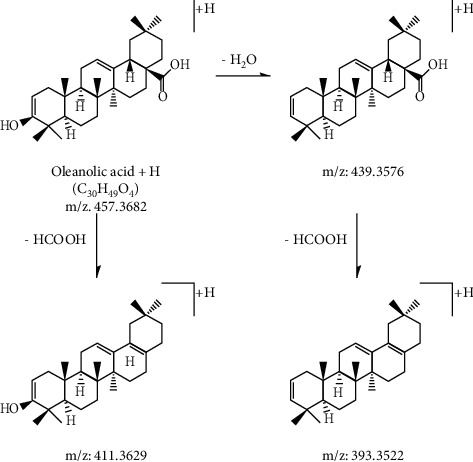
MS/MS fragmentation pathway of oleanolic acid aglycone in positive mode.

The chemical structures and fragmentation pathways of the hederagenin and oleanolic acid aglycones are illustrated in Figures 2 and 3, respectively.

#### 3.1.2. Sugar Chains

The sugar chains of triterpenoid saponins generally substitute at C‐3 and/or C‐28 position(s) of an aglycone. The common monosaccharide moieties of the sugar chains were glucopyranosyl (Glc), rhamnopyranosyl (Rha), arabinopyranosyl (Ara), and glucuronopyranosyl (Glu).

The composition of sugar chains can be inferred in the positive ion mode using the characteristic fragment ions, specifically as follows: the loss of Glc is 162 Da, Rha is 146 Da, Ara is 132 Da, and Glu is 176 Da. The sugar moieties at C‐3 and C‐28 were eliminated successively from C‐3 to C‐28 and from end to inner [12].

In the negative ion mode, the typical solvent adducts ion [M+HCOO]^−^ and deprotonated ion [M‐H]− can be usually observed, which provides the molecular mass and chemical formula of a compound. Typically, the sugar chain at C‐28 tends to be completely eliminated; then, the positions and composition of oligosaccharides chains can be readily differentiated and followed by an abundant fragment ion as a base peak [12].

### 3.2. Characterization of Authentic Compounds

To clarify MS fragmentation patterns of triterpene saponins, two authentic compounds including *α*-hederin and hederacoside C were studied by UHPLC-Q-TOF-MS/MS.

*α*-hederin (*T*_R_ = 20.41) showed a deprotonated molecular ion [M‐H]− at *m*/*z* 749.4473 in the negative mode and a pseudomolecular ion [M+H]^+^ at *m*/*z* 751.4633 in the positive mode.

At *T*_R_ = 12.49, hederacoside C yielded [M+H]^+^ ion at *m*/*z* 1221.6313 and [M‐H]^−^ ion at *m*/*z* 1219.6110 in the positive and negative modes, respectively.

**Figure 4:**
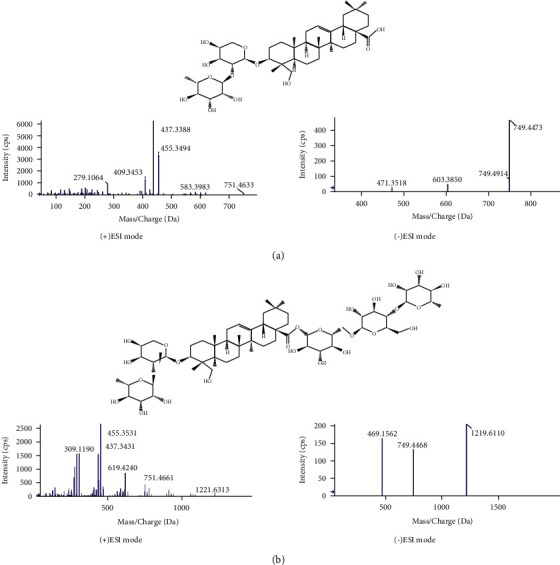
Typical MS and MS/MS spectra in positive and negative electrospray ionization modes of (a) *α*-hederin and (b) hederacoside C.

The major fragment ions observed in the mass spectra of the two triterpene saponins are summarized in Table 1. The typical MS and MS/MS spectra of *α*-hederin and hederacoside C are shown in Figure 4.

### 3.3. Structural Characterization of Triterpene Saponins

**Figure 5:**
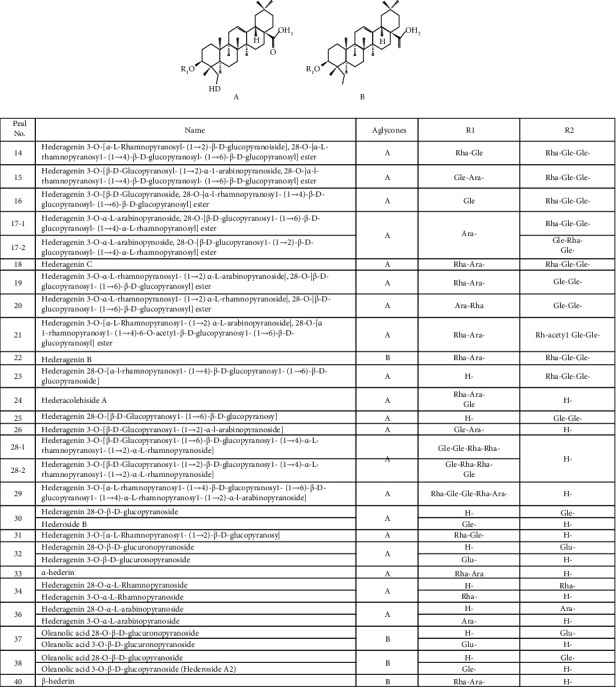
Chemical structures of identified triterpene saponins in *H. helix.*

Based on the earlier strategy, 24 triterpene saponins were tentatively identified and characterized from the *H. helix* extract. The chemical structures are illustrated in Figure 5, and the MS data are listed in Table 2.

At *T*_R_ = 11.11, in the negative mode, compound 14 yielded an [M+HCOO]^−^ ion at *m*/*z* 1295.6279 and provided fragment ions at *m*/*z* 779 and 469 corresponding to the loss of 2 Glc and 1 Rha at C-28, and a Rha-Glc sugar chain at C-3 of the hederagenin aglycone. Hence, compound 14 was hederagenin 3-O-[*α*-L-rhamnopyranosyl-(1⟶2)-*β*-D-glucopyranoside], 28-O-[*α*-L-rhamnopyranosyl-(1⟶4)-*β*-D-glucopyranosyl-(1⟶6)-*β*-D-glucopyranosyl] ester.

Compound 16 (*T*_R_ = 11.31 min) yielded an [M+HCOO]^−^ ion at *m*/*z* 1149.5707 in the negative mode, primarily fragmented into ions at *m*/*z* 633 and 469, indicating hederagenin aglycone lost a Rha-Glc-Glc sugar chain at C-28, and a Glc at C-3. Thus, compound 16 was conditionally characterized as hederagenin 3-O-*β*-D-glucopyranoside, 28-O-[*α*-L-rhamnopyranosyl-(1⟶4)-*β*-D-glucopyranosyl-(1⟶6)-*β*-D-glucopyranosyl] ester.

**Figure 6:**
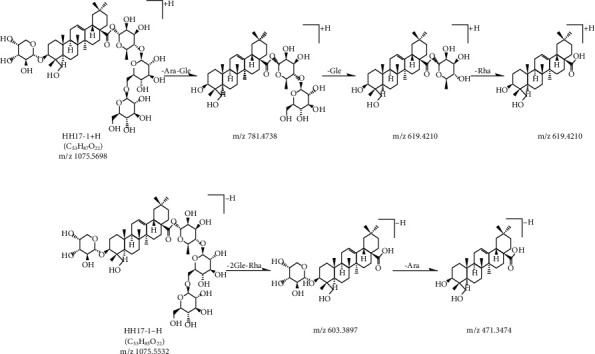
MS/MS fragmentation pathway of hederagenin 3-O*-α-*L*-*arabinopyranoside, 28*-*O-[*β*-D-glucopyranosyl-(1⟶6)-*β*-D-glucopyranosyl-(1⟶4)-*α*-L-rhamnopyranosyl] ester in positive and negative modes.

**Figure 7:**
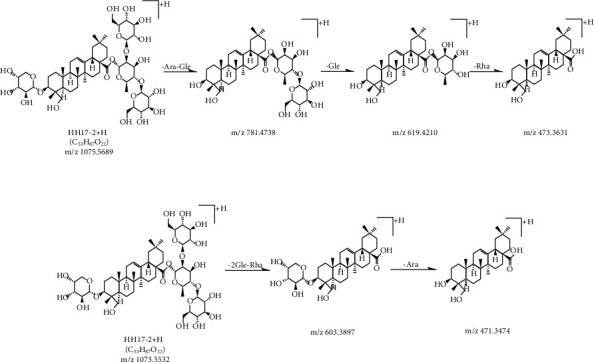
MS/MS fragmentation pathway of hederagenin 3-O*-α-*L*-*arabinopyranoside, 28*-*O-[*β*-D-glucopyranosyl-(1⟶2)-(*β*-D-glucopyranosyl-(1⟶4))-*α*-L-rhamnopyranosyl] ester in positive and negative modes.

In the positive mode, the MS/MS spectra of compounds 17 (*T*_R_ = 12.43 min) and 20 (*T*_R_ = 13.05 min) exhibited identical pseudomolecular ions [M+H]^+^ at *m*/*z* 1075.5687 and 1075.5680, respectively, and produced identical aglycone ions at *m*/*z* 391, 409, 437, 455, and 473, which corresponded to hederagenin. The fragmentation of compound 17 primarily yielded daughter ions at *m*/*z* 781, 619, and 473 because of the successive loss of Ara-Glc, Glc, and Rha. In addition, compound 17 produced a parent ion [M+HCOO]^−^ at *m*/*z* 1119.5508 in the negative mode, and fragmented into 603 and 469, indicating that the C‐28 position was substituted with 2 Glc and 1 Rha sugar chain. Consequently, compound 17 was characterized as hederagenin 3-O-*α*-L-arabinopyranoside, 28-O-[*β*-D-glucopyranosyl-(1⟶6)-*β*-D-glucopyranosyl-(1⟶4)-*α*-L-rhamnopyranosyl] ester or hederagenin 3-O-*α*-L-arabinopyranoside, 28-O-[*β*-D-glucopyranosyl-(1⟶2)-(*β*-D-glucopyranosyl-(1⟶4))-*α*-L-rhamnopyranosyl] ester. These were explored as two new structures, and the MS/MS fragmentation pathways of compound 17 are illustrated in Figures 6 and 7. However, fragmentation of [M+H]^+^ from compound 20 formed daughter ions at *m*/*z* 943, 797, 635, and 473, which corresponded to the sequential elimination of Ara, Rha, and 2 Glc, respectively. In the negative mode, [M-H]− ion at *m*/*z* 1073.5454 was fragmented to 749, indicating that the C‐28 position was substituted with the 2 Glc sugar chain. Hence, compound 20 was hederagenin 3-O-[*α*-L-arabinopyranosyl-(1⟶2)-*α*-L-rhamnopyranoside], 28-[*β*-D-glucopyranosyl-(1⟶6)-*β*-D-glucopyranosyl] ester.

The identified compounds 18 (*T*_R_ = 12.37 min) and 29 (*T*_R_ = 17.74 min) exhibited pseudomolecular ions [M+H]^+^ at *m*/*z* 1221.6233 and 1221.6303 in the positive mode, respectively, which fragmented in different manners. The fragmentation of these two saponins exhibited aglycone ions at *m*/*z* 473, 455, and 437, which corresponded to hederagenin. Comparing the MS/MS spectra and retention time information with the reference standards, compound 18 was undoubtedly determined as hederacoside C. Besides, under the negative mode, the parent ion of compound 29 was observed at *m*/*z* 1219.5990 and produced distinctive product ions at *m*/*z* 1073, 911, 749, 603, and 471 by the loss of sugar chain (Rha, Rha-Glc, Rha-Glc-Glc, Rha‐Glc-Glc‐Rha, and Rha‐Glc-Glc‐Rha-Ara) at C-3 of the aglycone. Consequently, compound 29 could be characterized as hederagenin 3-O-[*α*-L-rhamnopyranosyl-(1⟶4)-*β*-D-glucopyranosyl-(1⟶6)-*β*-D-glucopyranosyl-(1⟶4)-*α*-L-rhamnopyranosyl-(1⟶2)-*α*-L-arabinopyranoside].

At *T*_R_ = 12.63, in the negative mode, compound 19 yielded an [M+HCOO]^−^ ion at *m*/*z* 1119.5482 and provided fragment ions at *m*/*z* 749, 603, and 469 corresponding to the loss of 2 Glc at C-28 and a Rha-Ara sugar chain at C-3 of the hederagenin aglycone. Therefore, compound 19 was tentatively identified as hederagenin 3-O-[*α*-L-rhamnopyranosyl-(1⟶2)-*α*-L-arabinopyranoside], 28-O-[*β*-D-glucopyranosyl-(1⟶6)-*β*-D-glucopyranosyl] ester.

Compound 21 (*T*_R_ = 13.93 min) yielded an [M+HCOO]^−^ ion at *m*/*z* 1307.6264 in the negative mode, primarily fragmented into ions at *m*/*z* 791, 749, and 469, indicating that hederagenin aglycone C-28 lost an acetyl group in the Rha-Glc-Glc sugar chain, and C-3 lost a Rha-Ara sugar chain. Therefore, compound 21 was conditionally characterized as hederagenin 3-O-[*α*-L-Rhamnopyranosyl-(1⟶2)-*α*-L-arabinopyranoside], 28-O-[*α*-L-rhamnopyranosyl-(1⟶4)-6-O-acetyl-*β*-D-glucopyranosyl-(1⟶6)-*β*-D-glucopyranosyl] ester.

At *T*_R_ = 14.72, in the negative mode, compound 22 showed the formula of C_59_H_96_O_25_ ([M-H]^−^ at *m*/*z* 1203.6189) and provided fragment ions at *m*/*z* 733 and 455, corresponding to the loss of 2 Glc and 1 Rha at C-28, and a Rha-Ara sugar chain at C-3 of the oleanolic acid aglycone. Hence, compound 22 was identified as hederacoside B.

The MS spectra of compound 23 (*T*_R_ = 14.77 min) yielded a parent ion [M+HCOO]^−^ at *m*/*z* 987.5210 in the negative mode, primarily fragmented into an ion at *m*/*z* 471, indicating that the C-28 sugar chain contained a Rha and 2 Glc, and the aglycone corresponded to hederagenin. Thus, compound 23 was determined as hederagenin 28-O-[*α*-L-rhamnopyranosyl-(1⟶4)-*β*-D-glucopyranosyl-(1⟶6)-*β*-D-glucopyranoside].

Compound 24 (*T*_R_ = 15.42 min) yielded an [M+HCOO]^−^ ion at *m*/*z* 957.5047 in the negative mode, primarily fragmented into ions at *m*/*z* 749, 603, and 471, indicating the loss of Glc-Rha-Ara sugar chain at C-3 of hederagenin. Therefore, compound 24 was conditionally identified as hederagenin 3-O-[*α*-L-rhamnopyranosyl-(1⟶2)-(*β*-D-glucopyranosyl-(1⟶4))-*α*-L-arabinopyranoside] (hederacolchiside A).

Compound 25 showed the formula of C_42_H_68_O_14_ ([M+HCOO]^−^ at *m*/*z* 841.4599) and provided fragment ion at *m*/*z* 471, corresponding to the loss of 2 Glc at C-28 of the hederagenin aglycone. Hence, compound 25 was tentatively characterized as hederagenin 28-O-[*β*-D-glucopyranosyl-(1⟶6)-*β*-D-glucopyranoside].

The MS spectra of compound 26 (*T*_R_ = 15.78 min) yielded a parent ion [M+HCOO]^−^ at *m*/*z* 811.448, primarily fragmented into ions at *m*/*z* 603 and 471, indicating that the C-3 sugar chain contained a Glc and an Ara, and the aglycone corresponded to hederagenin. Thus, compound 26 was conditionally identified as hederagenin 3-O-[*β*-D-glucopyranosyl-(1⟶2)-*α*-L-arabinopyranoside].

**Figure 8:**
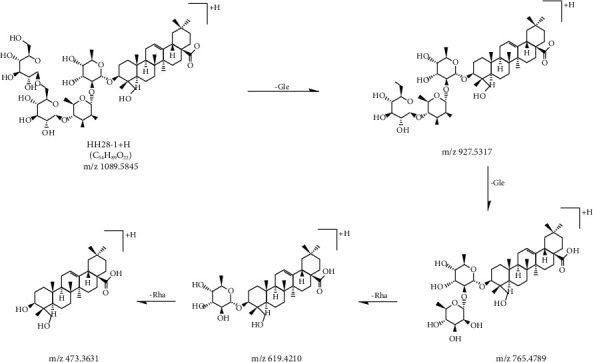
MS/MS fragmentation pathway of hederagenin 3-O-[*β*-D-glucopyranosyl-(1⟶6)-*β*-D-glucopyranosyl-(1⟶4)-*α*-L-rhamnopyranosyl-(1⟶2)-*α*-L-rhamnopyranoside] in positive mode.

**Figure 9:**
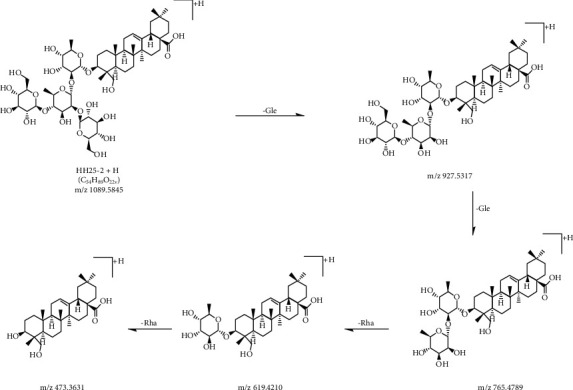
MS/MS fragmentation pathway of hederagenin 3-O-[*β*-D-glucopyranosyl-(1⟶2)-(*β*-D-glucopyranosyl-(1⟶4))-*α*-L-rhamnopyranosyl-(1⟶2)-*α*-L-rhamnopyranoside] in positive mode.

In the positive mode, the MS/MS spectra of compounds 28 (*T*_R_ = 17.68 min) exhibited pseudomolecular ions [M+H]^+^ at *m*/*z* 1089.5828, and produced aglycone ions at *m*/*z* 391, 409, 437, 455, and 473, which corresponded to hederagenin. The fragmentation of compound 28 primarily yielded daughter ions at *m*/*z* 927, 765, 619, and 473 because of the successive loss of Glc-Glc-Rha-Rha. By assembling all the fragmentation information compound 28, this compound was determined to be hederagenin 3-O-[*β*-D-glucopyranosyl-(1⟶6)-*β*-D-glucopyranosyl-(1⟶4)-*α*-L-rhamnopyranosyl-(1⟶2)-*α*-L-rhamnopyranoside] or hederagenin 3-O-[*β*-D-glucopyranosyl-(1⟶2)-(*β*-D-glucopyranosyl-(1⟶4))-*α*-L-rhamnopyranosyl-(1⟶2)-*α*-L-rhamnopyranoside]. They were investigated as two new saponin structures. The MS/MS fragmentation pathways of the structures of compound 28 are illustrated in Figures 8 and 9.

At *T*_R_ = 18.40, compound 30 showed the formula of C_36_H_58_O_9_ ([M+HCOO]^−^ at *m*/*z* 679.4052) and fragmented into ions at *m*/*z* 471, indicating that the C-28 or C-3 sugar chain contained a Glc, and the aglycone was hederagenin. Thus, compound 30 was determined as hederagenin 28-O-*β*-D-glucopyranoside or hederagenin 3-O-*β*-D-glucopyranoside (hederacoside B).

The MS spectra of compound 31 (*T*_R_ = 18.62 min) yielded a parent ion [M-H]^−^ at *m*/*z* 779.4586, primarily fragmented into ions at *m*/*z* 633 and 471, indicating the loss of a Rha-Glc sugar chain at C-3 of hederagenin. Hence, compound 31 was conditionally identified as hederagenin 3-O-[*α*-L-rhamnopyranosyl-(1⟶2)-*β*-D-glucopyranoside].

Compound 32 showed a deprotonated molecular ion [M‐H]^−^ at *m*/*z* 647.3793 at *T*_R_ = 19.50. The MS/MS spectra showed an ion peak at *m*/*z* 471, which indicated that at C-28 or C-3 of hederagenin contained a Glu. Therefore, compound 32 was predicted to be hederagenin 28-O-*β*-D-glucuronopyranoside or hederagenin 3-O-*β*-D-glucuronopyranoside.

Compound 33 (*T*_R_ = 20.30 min) yielded [M+H]^+^ ion at *m*/*z* 751.4609 [M‐H]^−^ ion at *m*/*z* 749.4489 in the positive and negative modes, respectively. Comparing the MS/MS information and retention time with the reference standard, compound 33 was unambiguously identified as *α*-hederin.

At *T*_R_ = 20.38, compound 34 showed the formula of C_36_H_58_O_8_ ([M+H]^+^ at *m*/*z* 619.4197). The fragment ions at *m*/*z* 391, 437, and 473 indicated the loss of a Rha at C-3 or C-28 of hederagenin aglycone. Thus, compound 34 was characterized as hederagenin 3-O-*α*-L-rhamnopyranoside or hederagenin 28-O-*α*-L-rhamnopyranoside.

The MS spectra of compound 36 (*T*_R_ = 21.16 min) yielded a parent ion [M+HCOO]^−^ at *m*/*z* 649.4963 and fragmented into 471, indicating the loss of an Ara at C-3 or C-28 of hederagenin. Consequently, compound 36 was identified as hederagenin 3-O-*α*-L-arabinopyranoside or hederagenin 28-O-*α*-L-arabinopyranoside.

Under the negative mode, compound 37 (*T*_R_ = 22.95 min) showed the diagnostic ion [M‐H]^−^ at *m*/*z* 631.3869 and a fragment ion at *m*/*z* 455, indicating that the C‐28 or C-3 position of oleanolic acid aglycone was substituted with a Glu. Thus, compound 37 was tentatively determined as oleanolic acid 28-O-*β*-D-glucuronopyranoside or oleanolic acid 3-O-*β*-D-glucuronopyranoside.

Compound 38 (*T*_R_ = 23.33 min) yielded an [M+HCOO]^−^ ion at *m*/*z* 663.4114 in the negative mode, primarily fragmented into an ion at *m*/*z* 455, meaning that the C-28 or C-3 sugar chain contained a Glc, and the aglycone corresponded to oleanolic acid. Hence, compound 38 was characterized as oleanolic acid 28-O-*β*-D-glucopyranoside or oleanolic acid 3-O-*β*-D-glucopyranoside (hederacoside A2).

The MS spectra of compound 40 (*T*_R_ = 24.03 min) yielded a pseudomolecular ion [M‐H]^−^ at *m*/*z* 733.4518. Under the negative mode, the diagnostic ions at *m*/*z* 587 and 455 indicated that in compound 40, the C‐28 position of oleanolic acid was substituted with a Rha, and the C-3 position of the aglycone contained an Ara, or the product ion represented for the loss of sugar chain Rha‐Ara at C-3 position only. Therefore, compound 40 was predicted to be oleanolic acid 3-O-*α*-L-rhamnopyranosyl-(1⟶2)-*α*-L-arabinopyranoside (*β*-hederin).

At *T*_R_ = 25.24, compound 42 showed the formula of C_30_H_48_O_4_ ([M-H]^−^ at *m*/*z* 471.3488) and fragmented into ion at *m*/*z* 393. Comparing the MS/MS spectrum and data with the result of Hai-Long et al. [26], compound 42 was characterized as hederagenin.

### 3.4. Structural Characterization of Flavonoids and Flavonoid Glycosides

In the positive mode, compounds 4, 5, 7, and 8 were identified as rutin (*m*/*z* 611.1623), quercetin (*m*/*z* 303.0504), kaempferol-3-O-rutinoside (*m*/*z* 595.1670), and kaempferol (*m*/*z* 287.0557), respectively, confirmed by MS and MS/MS data comparison with the results of Renu and Brijesh [27].

**Figure 10:**
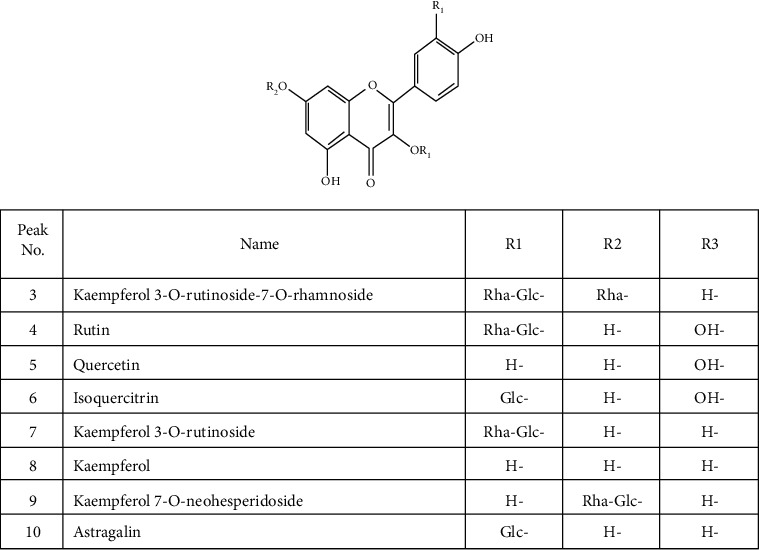
Chemical structures of flavonoids and flavonoid glycosides in *H. helix*.

In the negative mode, at *T*_R_ = 6.46, compound 3 exhibited an [M-H]^−^ ion at *m*/*z* 739.2050, and fragmented into 593 and 285, due to the elimination of a rhamnosyl group and a rutinosyl group, respectively. Therefore, compound 3 was identified as kaempferol 3-O-rutinoside-7-O-rhamnoside. Compound 6 yielded [M-H]^−^ ion at *m*/*z* 463.0897. Comparing the MS/MS data with Linling et al.'s publication [28], compound 6 was characterized as isoquercitrin. At *T*_R_ = 7.98, compound 10 showed deprotonated molecular ion [M-H]^−^ at *m*/*z* 447.0946 and produced a characteristic fragment ion at *m*/*z* 285, corresponding to the characteristic loss of a glucosyl group. Hence, compound 10 was determined as astragalin. All of the chemical structures of identified flavonoids are presented in Figure 10.

## 4. Conclusions

In the present work, by applying UHPLC‐Q‐TOF‐MS/MS in both positive and negative electrospray ionization modes as an efficient analytical method, the chemical constituents of *H. helix* could be rapidly discovered and identified in a single sample injection. As a result, 46 phytochemicals including 24 triterpene saponins were characterized, and four of which have yet been published before. UHPLC‐Q‐TOF‐MS/MS serves as a powerful analytical method for finding and instructing new phytochemical structures. Furthermore, the phytochemical profile result provides a base for quality control of *H. helix* raw materials. It also propels the medicinal application of this plant base on the metabolomic profiling of triterpene saponins.

## Figures and Tables

**Table 1:** The MS/MS data of standard compounds.

*T *_R_ (min)	Formula	Name	[M-H]^−^ found at mass	[M-H]^−^ error (ppm)	MS/MS fragment ions in negative mode	[M+H]^+^ found at mass	[M+H]^+^ error (ppm)	MS/MS fragment ions in positive mode
12.49	C_59_H_96_O_26_	Hederacoside C	1219.6110	−0.40	749.4487	1221.6313	3.68	1075.5653 943.5211 797.4660 635.4156 473.3614 455.3492 437.3389 427.3564 409.3441 391.3317

20.41	C_41_H_66_O_12_	*α*-hederin	749.4473	−0.16	603.3907 471.3473	751.4634	0.13	605.4065 473.3609 455.3494 437.338 427.3567 409.3457 391.3351

**Table 2:** Chemical constituents of *Hedera helix* characterized by UHPLC-Q-TOF-MS/MS.

Peak no.	*T*_R_ (min)	Formula	Chemical name	ESI mode	Error (ppm)	Exact mass	Found at mass	MS/MS
1	5.20	C_7_H_12_O_6_	(−)-Quinic acid	−	−2.43	191.0556	191.0551	173.0467 [M-H-H_2_O]^−^

2	5.21	C_16_H_18_O_9_	Chlorogenic acid	−	2.38	353.0873	353.0881	191.0548 [M-H-C_9_H_6_O_3_]^−^ 179.0336 [M-H-C_7_H_10_O_5_]^−^ 135.0443 [M-H-C_8_H_10_O_7_]^−^

3	6.46	C_33_H_40_O_19_	Kaempferol 3-O-rutinoside-7-O-rhamnoside	−	−4.82	739.2086	739.2050	593.1431 [M-H-C_6_H_10_O_4_]^−^ 285.0409 [M-H-C_18_H_30_O_13_]^−^

4	6.83	C_27_H_30_O_16_	Rutin	+	1.78	611.1612	611.1623	465.1035 [M+H-C_6_H_10_O_4_]^+^ 303.0501 [M+H-C_12_H_20_O_9_]^+^ 147.0661 [M+H-C_18_H_24_O_14_]^+^ 129.0551 [M+H-C_18_H_26_O_15_]^+^

5	6.84	C_15_H_10_O_7_	Quercetin	+	−0.25	303.0505	303.0504	257.0463 [M+H-CH_2_O_2_]^+^ 229.0507 [M+H-C_2_H_2_O_3_]^+^ 201.0594 [M+HC_3_H_2_O_4_]^+^ 165.0200 [M+H-C_7_H_6_O_3_]^+^ 153.0201 [M+H-C_8_H_6_O_3_]^+^ 137.0228 [M+H-C_8_H_6_O_4_]^+^

6	7.20	C_21_H_20_O_12_	Isoquercitrin	−	4.42	463.0877	463.0897	301.0332 [M-H-C_6_H_10_O_5_]^−^ 271.0227 [M-H-C_7_H_12_O_6_]^−^ 255.0276 [M-H-C_7_H_12_O_7_]^−^ 243.0286 [M-H-C_8_H_12_O_7_]^−^ 151.0036 [M-H-C_14_H_16_O_8_]^−^

7	7.50	C_27_H_30_O_15_	Kaempferol 3-O-rutinoside	+	1.18	595.1663	595.1670	449.1105 [M+H-C_6_H_10_O_4_]^+^ 287.0559 [M+H-C_12_H_20_O_9_]^+^ 147.0660 [M+H-C_18_H_24_O_13_]^+^ 129.0554 [M+H-C_17_H_22_O_15_]^+^

8	7.51	C_15_H_10_O_6_	Kaempferol	+	0.47	287.0556	287.0557	165.0204 [M+H-C_7_H_6_O_2_]^+^ 153.0197 [M+H-C_8_H_6_O_2_]^+^ 121.0273 [M+H-C_8_H_6_O_4_]^+^

9	7.55	C_27_H_30_O_15_	Kaempferol 7-O-neohesperidoside	−	1.43	593.1507	593.1515	285.0375 [M-H-C_12_H_20_O_9_]^−^ 151.0040 [M-H-C_20_H_26_O_11_]^−^

10	7.98	C_21_H_20_O_11_	Astragalin	−	4.16	447.0927	447.0946	285.0378 [M-H-C_6_H_10_O_5_]^−^ 255.0279 [M-H-C_7_H_12_O_6_]^−^

11	8.01	C_25_H_24_O_12_	Cynarin	−	−1.08	515.1190	515.1184	353.0837 [M-H-C_9_H_6_O_3_]^−^ 335.0746 [M-H-C_9_H_8_O_4_]^−^ 191.0535 [M-H-C_18_H_12_O_6_]^−^ 179.0327 [M-H-C_16_H_16_O_8_]^−^ 161.0230 [M-H-C_18_H_10_O_8_]^−^ 135.0438 [M-H-C_17_H_16_O_10_]^−^ 111.0441 [M-H-C_19_H_16_O_10_]^−^

12	8.59	C_25_H_24_O_12_	Isochlorogenic acid b	−	0.48	515.1190	515.1192	353.0850 [M-H-C_9_H_6_O_3_]^−^ 191.0541 [M-H-C_18_H_12_O_6_]^−^ 179.0332 [M-H-C_16_H_16_O_8_]^−^ 161.0232 [M-H-C_18_H_10_O_8_]^−^ 137.0227 [M-H-C_17_H_14_O_10_]^−^ 135.0442 [M-H-C_17_H_16_O_10_]^−^

13	8.63	C_9_H_16_O_4_	Azelaic acid	−	−0.72	187.0970	187.0969	125.0960 [M-H-CH_2_O_3_]^−^ 97.0643 [M-H-C_3_H_6_O_3_]^−^

14	11.11	C_60_H_98_O_27_	Hederagenin 3-O-[*α*-L-rhamnopyranosyl-(1⟶2)-*β*-D-glucopyranoside], 28-O-[*α*-L-rhamnopyranosyl-(1⟶4)-*β*-D-glucopyranosyl-(1⟶6)-*β*-D-glucopyranosyl] ester	−	0.54	1295.6272	1295.6279	1249.6105 [M-H]^−^ 779.4505 [M-H-C_18_H_30_O_14_]^−^ 469.1492 [M-H-C_30_H_52_O_23_]^−^

15	11.15	C_59_H_96_O_27_	Hederagenin 3-O-[*β*-D-glucopyranosyl-(1⟶2)-*α*-L-arabinopyranoside], 28-O-[*α*-L-rhamnopyranosyl-(1⟶4)-*β*-D-glucopyranosyl-(1⟶6)-*β*-D-glucopyranosyl] ester	−	1.05	1235.6061	1235.6074	765.4362 [M-H-C_18_H_30_O_14_]^−^ 469.1597 [M-H-C_29_H_50_O_23_]^−^

16	11.31	C_54_H_88_O_23_	Hederagenin 3-O-*β*-D-glucopyranoside, 28-O-[*α*-L-rhamnopyranosyl-(1⟶4)-*β*-D-glucopyranosyl-(1⟶6)-*β*-D-glucopyranosyl] ester	−	1.22	1149.5693	1149.5707	1103.5582 [M-H]^−^ 633.3921 [M-H-C_18_H_30_O_14_]^−^ 469.1606 [M-H-C_24_H_42_O_19_]^−^

17	12.43	C_53_H_86_O_22_	Hederagenin 3-O-*α*-L-arabinopyranoside, 28-O-[*β*-D-glucopyranosyl-(1⟶6)-*β*-D-glucopyranosyl-(1⟶4)-*α*-L-rhamnopyranosyl] ester or hederagenin 3-O-*α*-L-arabinopyranoside, 28-O-[*β*-D-glucopyranosyl-(1⟶2)-(*β*-D-glucopyranosyl-(1⟶4))-*α*-L-rhamnopyranosyl] ester	+	−0.19	1075.5689	1075.5687	781.4768 [M+H-C_11_H_18_O_9_]^+^ 619.4227 [M+H-C_17_H_28_O_14_]^+^ 473.3664 [M+H-C_23_H_38_O_18_]^+^ 455.3535 [M+H-C_23_H_40_O_19_]^+^ 437.3428 [M+H-C_23_H_42_O_20_]^+^ 409.3461 [M+H-C_24_H_42_O_21_]^+^ 391.3356 [M+H-C_24_H_44_O_22_]^+^

18	12.44	C_59_H_96_O_26_	Hederacoside C	+	−2.89	1221.6268	−	1075.5710 [M+H-C_6_H_10_O_4_]^+^ 943.5310 [M+H-C_11_H_18_O_8_]^+^ 797.4673 [M+H-C_17_H_28_O_12_]^+^ 635.4183 [M+H-C_23_H_38_O_17_]^+^ 473.3644 [M+H-C_29_H_48_O_22_]^+^ 455.3518 [M+H-C_29_H_50_O_23_]^+^ 437.3419 [M+H-C_29_H_52_O_24_]^+^ 427.3599 [M+H-C_30_H_50_O_24_]^+^ 409.3477 [M+H-C_30_H_52_O_25_]^+^ 391.3378 [M+H-C_30_H_54_O_26_]^+^

19	12.63	C_53_H_86_O_22_	Hederagenin 3-O-[*α*-L-rhamnopyranosyl-(1⟶2)-*α*-L-arabinopyranoside], 28-O-[*β*-D-glucopyranosyl-(1⟶6)-*β*-D-glucopyranosyl] ester	−	2.37	1119.5587	1119.5614	1073.5461 [M-H]^−^ 749.4439 [M-H-C_12_H_20_O_10_]^−^ 603.3906 [M-H-C_18_H_30_O_14_]^−^ 469.1536 [M-H-C_23_H_40_O_18_]^−^

20	13.05	C_53_H_86_O_22_	Hederagenin 3-O-[*β*-D-glucopyranosyl-(1⟶4)-*α*-L-rhamnopyranosyl-(1⟶2)-*α*-L-arabinopyranoside], 28-O-*β*-D-glucopyranosyl ester	+	−0.84	1075.5689	1075.5680	943.5316 [M+H-C_5_H_8_O_4_]^+^ 797.4678 [M+H-C_11_H_18_O_8_]^+^ 635.4170 [M+H-C_17_H_28_O_13_]^+^ 473.3622 [M+H-C_23_H_38_O_18_]^+^ 455.3528 [M+H-C_23_H_40_O_19_]^+^ 437.3424 [M+H-C_23_H_42_O_20_]^+^ 427.3555 [M+H-C_24_H_40_O_20_]^+^ 409.3468 [M+H-C_24_H_42_O_21_]^+^ 391.3371 [M+H-C_24_H_44_O_22_]^+^

21	13.93	C_61_H_98_O_27_	Hederagenin 3-O-[*α*-L-rhamnopyranosyl-(1⟶2)-*α*-L-arabinopyranoside], 28-O-[*α*-L-rhamnopyranosyl-(1⟶4)-6-O-acetyl-*β*-D-glucopyranosyl-(1⟶6)-*β*-D-glucopyranosyl] ester	−	−0.61	1307.6272	1307.6264	1261.6083 [M-H]^−^ 791.4544 [M-H-C_18_H_30_O_14_]^−^ 749.4448 [M-H-C_20_H_32_O_15_]^−^ 469.1563 [M-H-C_31_H_52_O_23_]^−^

22	14.72	C_59_H_96_O_25_	Hederacoside B	−	2.20	1203.6163	1203.6189	733.4469 [M-H-C_18_H_30_O_14_]^−^ 455.3473 [M-H-C_29_H_48_O_22_]^−^

23	14.77	C_48_H_78_O_18_	Hederagenin 28-O-[*α*-L-rhamnopyranosyl-(1⟶4)-*β*-D-glucopyranosyl-(1⟶6)-*β*-D-glucopyranoside]	−	4.58	987.5165	987.5210	941.5034 [M-H]^−^ 471.3436 [M-H-C_18_H_30_O_14_]^−^ 469.1547 [M-H-C_18_H_32_O_14_]^−^

24	15.42	C_47_H_76_O_17_	Hederagenin 3-O-[*α*-L-rhamnopyranosyl-(1⟶2)-(*β*-D-glucopyranosyl-(1⟶4))-*α*-L-arabinopyranoside] (hederacolchiside A)	−	−1.25	957.5059	957.5047	911.4947 [M-H]^−^ 749.4425 [M-H-C_6_H_10_O_5_]^−^ 603.3794 [M-H-C_12_H_20_O_9_]^−^ 471.3381 [M-H-C_17_H_28_O_13_]^−^

25	15.55	C_42_H_68_O_14_	Hederagenin 28-O-[*β*-D-glucopyranosyl-(1⟶2)-*β*-D-glucopyranoside]	−	1.54	841.4586	841.4599	795.4481 [M-H]^−^ 471.3440 [M-H-C_12_H_20_O_10_]^−^

26	15.78	C_41_H_66_O_13_	Hederagenin 3-O-[*β*-D-glucopyranosyl-(1⟶2)-*α*-L-arabinopyranoside]	−	0.12	811.4480	811.4481	471.2603 [M-H-C_11_H_18_O_9_]^−^ 603.3852 [M-H-C_6_H_10_O_5_]^−^ 765.4429 [M-H]^−^

27	17.05	C_29_H_42_O_6_	Kendomycin	−	−0.24	485.2903	485.2902	467.2769 [M-H-H_2_O]^−^ 439.2825 [M-H-C_2_H_6_O]^−^ 423.2866 [M-H-C_2_H_6_O_2_]^−^ 409.2718 [M-H-C_3_H_8_O_2_]^−^

28	17.68	C_54_H_88_O_22_	Hederagenin 3-O-[*β*-D-glucopyranosyl-(1⟶6)-*β*-D-glucopyranosyl-(1⟶4)-*α*-L-rhamnopyranosyl-(1⟶2)-*α*-L-rhamnopyranoside] or hederagenin 3-O-[*β*-D-glucopyranosyl-(1⟶2)-(*β*-D-glucopyranosyl-(1⟶4))-*α*-L-rhamnopyranosyl-(1⟶2)-*α*-L-rhamnopyranoside]	+	−1.65	1089.5846	1089.5828	927.5329 [M-H-C_6_H_10_O_5_]^−^ 765.4851 [M-H-C_12_H_20_O_10_]^−^ 619.4226 [M-H-C_18_H_30_O_14_]^−^ 473.3637 [M-H-C_24_H_40_O_18_]^−^ 455.3556 [M-H-C_24_H_42_O_19_]^−^ 437.3478 [M-H-C_24_H_44_O_20_]^−^ 409.3478 [M-H-C_25_H_44_O_21_]^−^ 391.3277 [M-H-C_25_H_46_O_22_]^−^

29	17.74	C_59_H_96_O_26_	Hederagenin 3-O-[*α*-L-rhamnopyranosyl-(1⟶4)-*β*-D-glucopyranosyl-(1⟶6)-*β*-D-glucopyranosyl-(1⟶4)-*α*-L-rhamnopyranosyl-(1⟶2)-*α*-L-arabinopyranoside]	−	−0.87	1219.6112	1219.6101	1073.5520 [M-H-C_6_H_10_O_4_]^−^ 749.4446 [M-H-C_18_H_30_O_14_]^−^ 603.3801 [M-H-C_24_H_40_O_18_]^−^ 471.3384 [M-H-C_29_H_48_O_22_]^−^

30	18.40	C_36_H_58_O_9_	Hederagenin 28-O-*β*-D-glucopyranoside or hederagenin 3-O-*β*-D-glucopyranoside (hederacoside B)	−	−0.79	679.4057	679.4052	633.3950 [M-H]^−^ 471.3433 [M-H-C_6_H_10_O_5_]^−^

31	18.62	C_42_H_68_O_13_	Hederagenin 3-O-[*α*-L-rhamnopyranosyl-(1⟶2)-*β*-D-glucopyranoside]	−	0.51	779.4582	779.4586	633.4022 [M-H-C_6_H_10_O_4_]^−^ 471.3355 [M-H-C_12_H_20_O_9_]^−^

32	19.50	C_36_H_56_O_10_	Hederagenin 28-O-*β*-D-glucuronopyranoside or hederagenin 3-O-*β*-D-glucuronopyranoside (HN saponin K)	−	−0.35	647.3795	647.3793	471.3395 [M-H-C_6_H_8_O_6_]^−^

33	20.30	C_41_H_66_O_12_	*α*-Hederin	−	1.73	749.4476	749.4489	603.3763 [M-H-C_6_H_10_O_4_]^−^ 471.3498 [M-H-C_11_H_18_O_8_]^−^

34	20.38	C_36_H_58_O_8_	Hederagenin 3-O-*α*-L-rhamnopyranoside or hederagenin 28-O-*α*-L-rhamnopyranoside	+	−2.10	619.4210	619.4197	473.3735 [M-H-C_6_H_10_O_4_]^−^ 455.3526 [M-H-C_6_H_12_O_5_]^−^ 437.3413 [M-H-C_6_H_14_O_6_]^−^ 409.3468 [M-H-C_7_H_14_O_7_]^−^ 391.3366 [M-H-C_7_H_16_O_8_]^−^

35	20.82	C_30_H_48_O_5_	Caulophyllogenin	−	−0.92	487.3424	487.3419	425.3407 [M-H-CH_2_O_3_]^−^ 423.3287 [M-H-CH_4_O_3_]^−^ 405.3127 [M-H-CH_6_O_4_]^−^ 393.3132 [M-H-C_2_H_6_O_4_]^−^

36	21.16	C_35_H_56_O_8_	Hederagenin 3-O-*α*-L-arabinopyranoside or hederagenin 28-O-*α*-L-arabinopyranoside	−	1.69	649.3952	649.3963	603.3823 [M-H]^−^ 471.3441 [M-H-C_5_H_8_O_4_]^−^

37	22.95	C_36_H_56_O_9_	Oleanolic acid 28-O-*β*-D-glucuronopyranoside or Oleanolic acid 3-O-*β*-D-glucuronopyranoside	−	3.63	631.3846	631.3869	455.3502 [M-H-C_6_H_8_O_6_]^−^

38	23.33	C_36_H_58_O_8_	Oleanolic acid 28-O-glucoside or oleanolic acid 3-O-glucoside (hederacoside A2)	−	0.87	663.4108	663.4114	617.4020 [M-H]^−^ 455.3488 [M-H-C_6_H_10_O_5_]^−^

39	23.78	C_18_H_30_O_3_	9S-hydroxy-10E,12Z,15Z-octadecatrienoic acid	−	3.51	293.2117	293.2127	275.1995 [M-H-H_2_O]^−^ 231.2092 [M-H-CH_2_O_3_]^−^ 221.1527 [M-H-C_3_H_4_O_2_]^−^ 183.1375 [M-H-C_7_H_10_O]^−^ 121.1010 [M-H-C_9_H_16_O_3_]^−^

40	24.03	C_41_H_66_O_11_	Oleanolic acid 3-O-*α*-L-arabinoside, 28-O-*α*-L-rhamnopyranosyl ester or oleanolic acid 3-O-*α*-L-rhamnopyranosyl-(1⟶2)-*α*-L-arabinopyranoside (*β*-hederin)	−	−1.21	733.4527	733.4518	587.3918 [M-H-C_6_H_10_O_4_]^−^ 455.3483 [M-H-C_11_H_18_O_8_]^−^

41	25.16	C_18_H_28_O_3_	9-Oxo-10E,12Z,15Z-octadecatrienoic acid	−	−2.47	291.1960	291.1953	197.1171 [M-H-C_7_H_10_]^−^ 185.1173 [M-H-C_8_H_10_]^−^ 121.1010 [M-H-C_9_H_14_O_3_]^−^

42	25.24	C_30_H_48_O_4_	Hederagenin	−	2.90	471.3474	471.3488	393.3153 [M-H-C_2_H_6_O_3_]^−^

43	26.31	C_30_H_46_O_4_	Hederagonic acid	+	2.05	471.3474	471.3484	453.3367 [M+H-H_2_O]^+^ 407.3302 [M+H-CH_4_O_3_]^+^ 389.3212 [M+H-CH_6_O_4_]^+^

44	27.29	C_18_H_30_O_3_	9-Oxo-10E,12Z-octadecadienoic acid	−	3.85	293.2117	293.2128	221.1532 [M-H-C_3_H_4_O_2_]^−^ 197.1171 [M-H-C_7_H_12_]^−^ 185.1160 [M-H-C_8_H_12_]^−^ 149.0962 [M-H-C_8_H_16_O_2_]^−^ 125.0958 [M-H-C_9_H_12_O_3_]^−^

45	32.56	C_16_H_32_O_3_	2-Hydroxypalmitic acid	−	−3.76	271.2273	271.2263	225.2200 [M-H-CH_2_O_2_]^−^

46	35.96	C_39_H_64_O_5_	Di-gamma-linolenic	+	−2.28	613.4832	613.4818	595.4736 [M+H-H_2_O]^+^ 539.4475 [M+H-C_4_H_10_O]^+^ 521.4385 [M+H-C_4_H_12_O_2_]^+^ 503.3767 [M+H-C_8_H_14_]^+^ 335.2586 [M+H-C_18_H_30_O_2_]^+^ 299.2366 [M+H-C_23_H_38_]^+^ 261.2219 [M+H-C_21_H_36_O_4_]^+^ 259.2075 [M+H-C_26_H_42_]^+^ 161.1322 [M+H-C_33_H_56_]^+^ 147.1164 [M+H-C_33_H_54_O]

## Data Availability

The data used to support the results of this study are included within the article. Any further information is available from authors upon request.
